# High-Sensitivity Identification of Micro-Voids at Thick Steel Shell–Concrete Interfaces Using Elastic Wave Analysis and Feature Attention Mechanisms

**DOI:** 10.3390/s26144428

**Published:** 2026-07-12

**Authors:** Yan Zhang, Siying Qu, Songhui Li, Yi Liu, Xunnan Liu

**Affiliations:** State Key Laboratory of Water Cycle and Water Security, China Institute of Water Resources and Hydropower Research, Beijing 100038, China; qusiying0820@163.com (S.Q.);

**Keywords:** steel–concrete composite structures, micro-interfacial voids, impact-echo, feature attention, multi-layer perceptron

## Abstract

The steel–concrete interface in steel–concrete composite structures is susceptible to interfacial void defects during both casting and service, posing a significant threat to structural load-bearing capacity. For early-stage micro-voids exceeding 2 mm in height, signal variations are weak and exhibit response characteristics similar to dense states, leading to feature ambiguity when using conventional criteria based on time-domain amplitude and attenuation or frequency-domain peak values and resulting in a high risk of missed detections. To address this limitation for early warning purposes, this study proposes a high-sensitivity identification method integrating an impact elastic wave response feature system with a feature-attention gated multi-layer perceptron (Feature-attention MLP). Based on full-scale model experiments from an engineering project, the temporal and spectral evolution patterns of impact elastic wave responses under varying dense conditions were analyzed. A comprehensive feature system, including time-domain statistical descriptors, spectral peaks, and sub-band energy distributions, was constructed, with Random Forest used for feature importance ranking and Top-K selection. An MLP classifier was then developed for automatic discrimination of dense states. A feature-level attention gating mechanism was introduced to enable adaptive weighting across feature dimensions, enhancing sensitive features while suppressing noise and structural variability. The final lightweight classifier contains 4052 trainable parameters, enabling rapid execution with an average CPU inference time of approximately 1.24 ms per sample. The average CPU inference time was approximately 1.24 ms per sample. Under the original train–validation split, the recall-prioritized operating point achieved a Void recall of 0.978 and a weighted F1-score of 0.780, accompanied by a non-negligible false-positive screening burden. Stratified five-fold internal validation yielded a balanced accuracy of 0.682 ± 0.021 and a Void recall of 0.845 ± 0.035 under the inner-validation-optimized threshold. These results demonstrate the preliminary potential of the proposed lightweight framework for engineering-oriented micro-void screening under the investigated full-scale conditions.

## 1. Introduction

Steel shell–concrete structures constitute a composite system in which concrete is filled within a steel shell. Acting as external confinement and a load-bearing framework, the steel shell facilitates synergistic interaction between the two materials, thereby fully exploiting their respective performance advantages. Such structures exhibit high load-bearing capacity, superior ductility, and construction efficiency and are widely applied in cross-sea passages, nuclear power protection facilities, and large-scale marine engineering projects [1,2,3]. A representative example is the immersed tunnel of the Shenzhen–Zhongshan Link. With a total underwater tunnel length of 6845 m and an immersed tube section of approximately 5035 m, this project adopts a steel shell–concrete structural scheme with a shell thickness of 44 mm, representing the largest steel shell–concrete immersed tunnel project worldwide to date [4]. Despite their favorable constructability and durability, underwater tunnels remain susceptible to structural response issues induced by complex hydrogeological environments during long-term service. Relevant research and engineering monitoring data indicate that challenges such as joint opening and closing, differential settlement, and long-term condition assessment necessitate robust structural health monitoring and service diagnosis systems to ensure full lifecycle performance [5,6]. Therefore, establishing an effective structural health monitoring and service condition evaluation framework is essential for safeguarding the lifecycle integrity of steel shell–concrete immersed tube structures.

However, the service performance of these structures depends critically on effective interaction at the steel shell–concrete interface. Influenced by factors such as casting processes and long-term loading, local interfacial defects, particularly voids, are prone to develop. Under cyclic loading, these interfacial voids exhibit propagative and cumulative behavior, weakening the composite strengthening effect between the steel shell and concrete. This degradation may induce stress redistribution, increased susceptibility to local buckling, and deterioration in durability, thereby compromising structural safety [7,8,9,10,11,12]. Existing model tests on sandwich-structured immersed tunnels (SSIT) indicate that, for steel shell structures with a thickness of 14 mm, the load-bearing capacity and stiffness of connectors decrease significantly when local void heights reach 10 mm. Furthermore, these studies explicitly recommend that void heights exceeding 5 mm should be avoided in structural design [13,14,15,16]. Consequently, early detection of micro-voids during the casting and operational stages of steel shell–concrete structures is of substantial engineering importance.

Interfacial voids between steel shells and concrete are typically highly concealed and difficult to observe directly. Common non-destructive testing (NDT) methods, including tap testing, infrared thermography, ground-penetrating radar, ultrasonic testing, neutron radiography, and impact elastic wave techniques [17,18,19,20,21,22], offer distinct advantages in engineering practice. However, they exhibit inherent limitations in complex inspection environments, such as detection through the 44-mm-thick steel shell of the Shenzhen–Zhongshan Link. For example, tap testing is predominantly qualitative; infrared thermography is limited by detection depth and environmental conditions; radar and ultrasonic methods are susceptible to shielding effects from steel components and on-site interference; and radiographic methods, although providing high resolution, suffer from poor field adaptability and safety concerns. Consequently, these techniques have limited capability in distinguishing early-stage or micro-scale voids. In contrast, detection approaches based on mechanical elastic waves are less constrained by the medium of the tested structure. With clear physical interpretation and multiple quantifiable parameters, such methods demonstrate greater potential for stable energy propagation in thick media, identification of concealed defects, and accurate quantitative evaluation.

From the perspective of technological evolution, elastic wave-based NDT has progressed from reliance on wave velocity and propagation time metrics, through impact-echo frequency-domain resonance criteria, to advanced time–frequency representations and intelligent identification paradigms. Early methodologies prioritized propagation time and wave velocity as primary indicators; notably, Whitehurst’s Soniscope concept established a foundation for assessing concrete quality via stress wave propagation characteristics. Subsequently, the pulse velocity method was standardized, culminating in regulatory frameworks such as ASTM C597 [23]. In the 1980s, Sansalone and Carino systematically proposed the Impact-Echo (IE) method, which employs short-duration impacts to excite transient stress waves and utilizes frequency-domain resonance information for defect identification, thereby facilitating engineering applications [24,25,26]. However, applying this principle to identify early-stage or micro-voids at thick steel shell–concrete interface presents significant limitations, as conventional signal analysis techniques are insufficiently sensitive. To address these challenges, researchers have adopted signal processing techniques including Short-Time Fourier Transform (STFT), Wavelet Transform (WT/CWT), and Empirical Mode Decomposition variants (EMD/EEMD/CEEMDAN) to enhance weak features, separate aliased modes, and improve interpretability [27,28,29,30]. These studies indicate that signal variations during micro-void formation are subtle and easily obscured by the complex responses induced by thick steel shell structures. The resulting changes in interface stiffness typically manifest as energy redistribution within localized frequency bands, modifications in secondary peak structures, or increased transient fluctuations, rather than as stable and significant shifts in dominant peak frequencies. Furthermore, dominant frequencies associated with different void depths may overlap, and the time–frequency characteristics of micro-voids often resemble those of dense states, leading to feature superposition. In thick steel shell–concrete composite structures, high-frequency impact-elastic-wave components are influenced not only by localized interface voids, but also by impact conditions, sensor coupling, interface transmission, structural boundaries, and propagation attenuation [31]. Therefore, fixed frequency filtering or single spectral parameters cannot reliably isolate defect-related attenuation signatures from other high-frequency response components. In addition, measurements are obtained at discrete inspection locations rather than as a spatially continuous field; conventional spatial filtering may therefore be insufficient to stably distinguish localized micro-void responses [32]. These limitations motivate the joint use of time-domain, frequency-domain, and energy-distribution features for micro-void identification.

Consequently, identifiable information related to micro-voids is not concentrated within a single time-domain or frequency-domain metric [33]; rather, it is distributed across time-domain statistical features, spectral peak structures, sub-band energy distributions, local time–frequency evolution patterns, and their interrelationships. Therefore, it is essential to construct a structured feature system derived from raw responses that captures variations in interface conditions. Moreover, adaptive adjustment of feature importance is required to identify potential structural changes embedded within high-dimensional feature spaces that may be obscured by subtle signal variations. In response, data-driven approaches based on feature learning, machine learning, and deep learning have been increasingly introduced. For example, the effectiveness of frameworks combining wavelet features with machine learning algorithms or Convolutional Neural Networks (CNNs) has been demonstrated in studies on bridge deck delamination [34,35]. Other studies have employed impact-acoustic signals with unsupervised learning to identify debonding and voids in concrete-filled steel tube structures [36], while additional research has developed void detection frameworks that balance computational efficiency and recognition performance by integrating acoustic features with ensemble learning methods [37]. Furthermore, attention mechanisms have been incorporated into elastic wave-based defect identification to enhance robustness and discriminative capability in noisy environments [38]. Simultaneously, interpretable attention-based prototype networks designed for small-sample guided wave damage identification provide methodological support for generating explainable outputs based on key evidence [39]. These studies demonstrate that integrating time–frequency features with deep learning can improve the automatic identification of complex signals to a certain extent. Beyond signal-based methods, recent SHM studies have increasingly adopted deep-vision architectures that integrate convolutional representations with Transformer backbones. Transformer-based high-resolution image segmentation [40], parallel Swin-CNN segmentation for concrete cracks [41], Cross-Shaped Window Transformer-based classification [42], and hybrid Vision Transformer-based micro-crack detection [43] have shown that the joint modeling of local detail and global contextual information can improve visual damage recognition. In these architectures, self-attention provides a mechanism for establishing non-local relationships among spatially separated image patches. Related vibration-based SHM studies have also investigated Transformer-based masked autoencoders for learning transferable representations from sensor signals, indicating the potential of attention-based models for capturing long-range dependencies in monitoring data [44]. However, achieving accurate identification of millimeter-scale voids in thick steel shell–concrete structures still requires further investigation into feature variability under identical dense states and feature similarity across different dense states while simultaneously improving identification accuracy and meeting practical engineering requirements such as minimizing missed detections.

In summary, this study proposes a high-sensitivity identification method for micro-voids at thick steel shell–concrete interfaces based on mechanical elastic waves and a feature attention mechanism. First, full-scale model experiments of thick steel shell–concrete structures were conducted using the Shenzhen–Zhongshan Link, currently the world’s largest underwater tunnel, to systematically analyze feature overlap and evolution patterns of impact response signals in both time and frequency domains. The analysis focused on representative interface conditions: dense, slight voids (2–3 mm), and severe voids (>3 mm). Second, sensitive indicators characterizing variations in interface states were extracted to construct a structured feature system. Third, a Multi-Layer Perceptron (MLP) model incorporating a feature attention mechanism was developed. Through adaptive feature-level weighting and nonlinear discriminative learning, this model enhances the extraction of weak features associated with micro-voids, thereby enabling high-sensitivity identification. Finally, the proposed method was validated using field measurement data from the Shenzhen–Zhongshan Link.

Unlike recent deep learning-based NDT approaches that primarily rely on end-to-end learning from raw signals, this study introduces a physics-informed structured feature system combined with a feature-attention MLP to enhance interpretability and improve sensitivity to weak micro-void signals in highly overlapping response conditions. It provides theoretical support and methodological guidance for intelligent inspection and early risk warning of micro-voids at the interfaces of thick steel shell–concrete structures.

## 2. Signal Feature Analysis

### 2.1. Full-Scale Model Experiment

**Figure 1:**
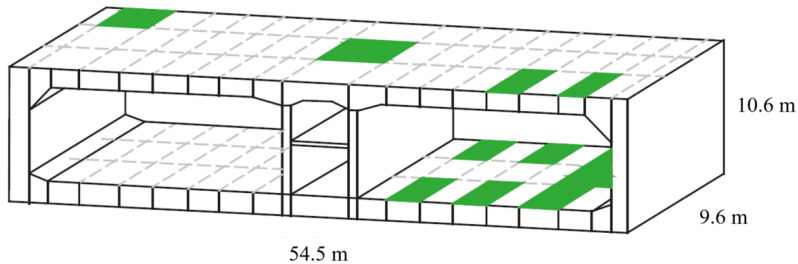
Schematic diagram of the overall structure of the full-scale model.

**Figure 2:**
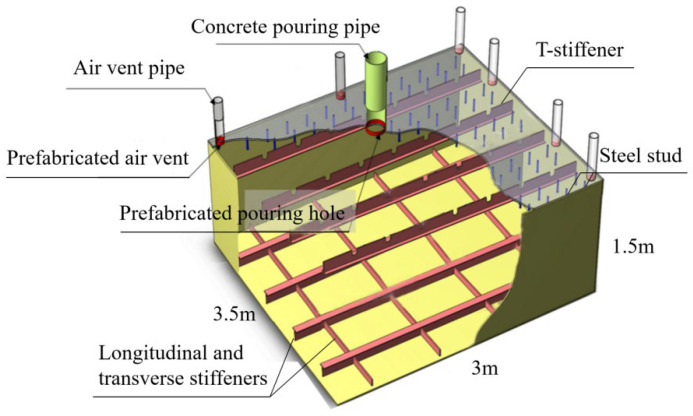
Schematic diagram of the compartment structure of the full-scale model.

This study employs a full-scale model of a steel shell–concrete immersed tunnel from the Shenzhen–Zhongshan Link. As illustrated in Figure 1, the model comprises 105 compartments with dimensions of 9.6 m in length, 54.5 m in width, and 10.6 m in height. The internal configuration of the compartments is shown in Figure 2. The outer steel shell was fabricated using Q345B steel with thicknesses of 14 mm and 44 mm, while the core concrete exhibits a compressive strength not less than 42.5 MPa. The model was fabricated, transported, and cast under conditions representative of actual engineering practice to replicate void defects that may arise during standard production and construction processes. Detailed procedures regarding tunnel configuration, self-compacting concrete casting techniques, grid-based inspection layouts, and cover-opening protocols for verification and ground truth calibration have been systematically reported in prior studies [45,46].

**Figure 3:**
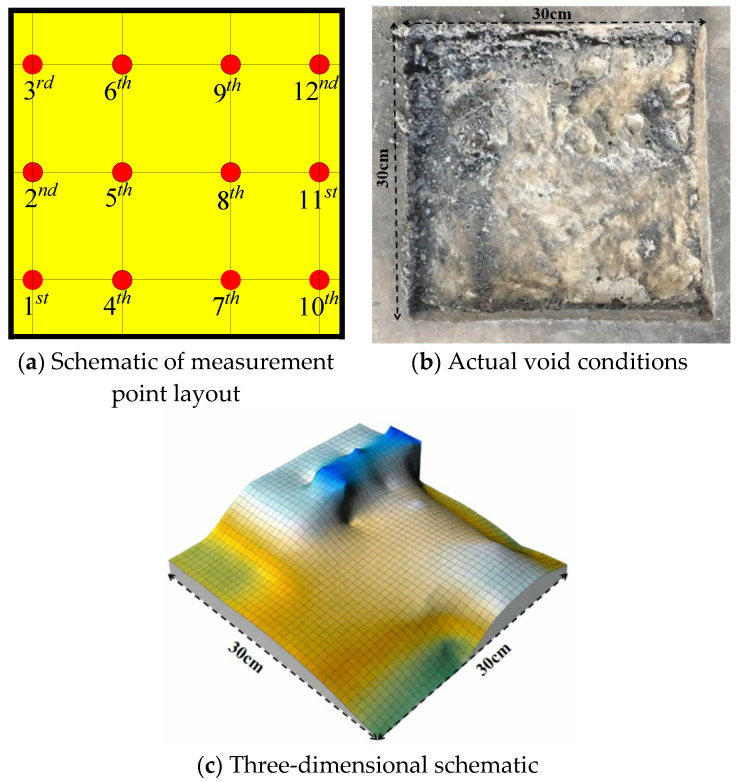
Layout of measurement points at cover-opening locations, actual void conditions, and three-dimensional schematic.

Data acquisition was conducted on a compartment-by-compartment basis using both single-point measurements and survey line methods. Selected compartments were subjected to cover-opening verification. Based on measured void depths in defective regions, the dataset was classified into three categories according to void depth, as summarized in Table 1. Figure 3 presents the layout of measurement points at cover-opening locations, the actual void conditions, and a three-dimensional schematic representation.

### 2.2. Signal Features

#### 2.2.1. Raw Signals

**Figure 4:**
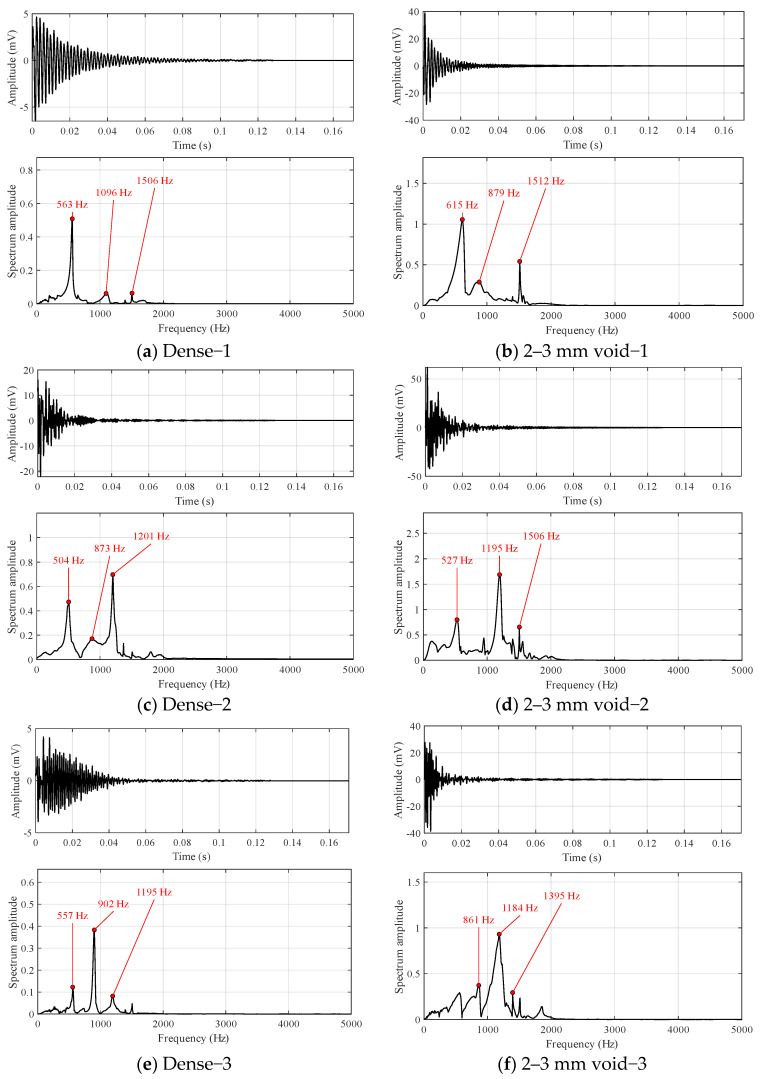

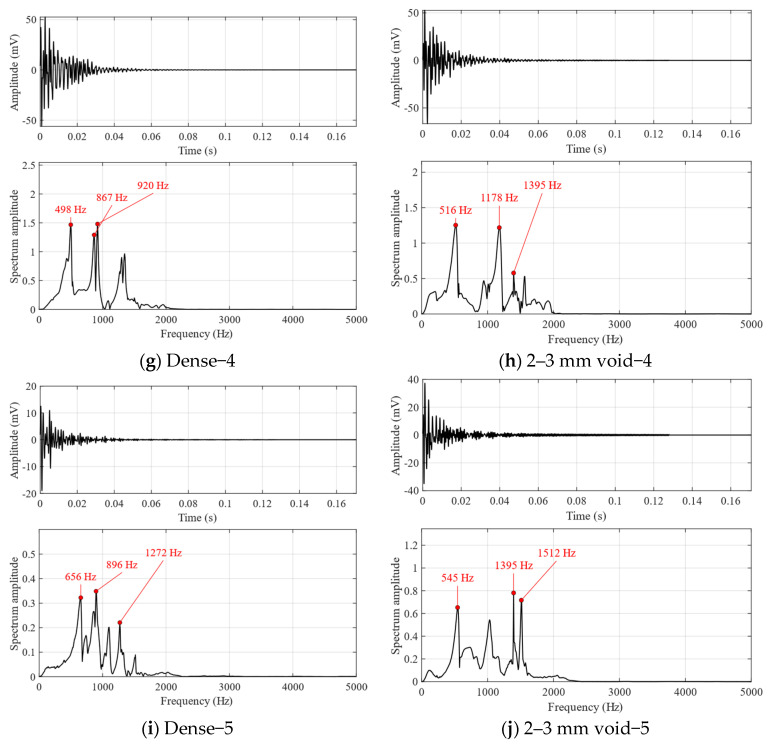
Comparison of time-domain and frequency-domain waveforms between dense regions and 2–3 mm void regions in steel-shelled concrete.

Analysis and classification of impact response signals from three groups with different density states indicate that signals within the same state exhibit significant variability, while signals across different states may display similar characteristics, revealing an inherent contradiction. Taking the dense state and the 2–3 mm void state as examples, Figure 4 shows their time-domain waveforms and frequency-domain spectra obtained via Fourier transform. The following observations are noted: First, dense-state responses can be categorized into five distinct types with clearly differentiated spectral characteristics. For example, Figure 4a exhibits a single dominant frequency, Figure 4c shows two dominant frequencies, Figure 4e presents one dominant frequency accompanied by multiple pronounced peaks, and Figure 4g,f display three or more spectral peaks. Correspondingly, time-domain signals differ in attenuation patterns, amplitude levels, and other characteristics. Thus, no specific dense state corresponds uniquely to a fixed time–frequency signature. Second, substantial overlap exists in time–frequency features across different states. Nearly all typical waveform or spectral patterns observed in the dense state (Figure 4) are also present in samples with 2–3 mm voids. This indicates that early-stage micro-void responses have not yet formed independent patterns but instead appear as localized perturbations and gradual evolution of the dense-state response. Consequently, these sample types exhibit significant overlap in low-dimensional discriminant space.

Further comparison shows that differences across density states are primarily reflected in localized time–frequency details. For example, Figure 4b,g exhibit strong similarity in the time domain, with comparable amplitude ranges and attenuation durations, yet differ in spectral characteristics. Within the 500–1500 Hz range, differences are observed in peak distribution, relative prominence of primary and secondary peaks, and high-frequency attenuation behavior. Conversely, Figure 4a,b display similar spectral patterns but significantly differ in time-domain responses: Figure 4a is relatively smooth with weak transient fluctuations, whereas Figure 4b shows pronounced local oscillations and extended attenuation tails. Overall, distinctions between dense and micro-void conditions are dispersed, localized, and inconsistent rather than concentrated in a single feature. Therefore, reliance on individual time-domain or frequency-domain indicators is insufficient, necessitating integrated multi-domain feature analysis.

#### 2.2.2. Sensitive Parameter Extraction and Feature System Construction

The above analysis indicates that impact responses associated with micro-voids at steel–concrete interfaces are characterized by weak discriminability, high intra-class variability, and strong overlap with dense-state spectra. Accordingly, this study extracts signal features from three dimensions—time-domain statistics, spectral peak structure, and sub-band energy redistribution—focusing on latent information embedded in waveform signals.

(1) Time-domain statistical features

Micro-voids may manifest in the time domain as localized energy fluctuations, extended attenuation tails, and variations in positive/negative peak amplitudes. To quantify these effects, a set of time-domain statistical parameters is introduced to characterize overall fluctuation intensity and signal complexity. The standard deviation (STD) represents signal dispersion and overall variability:

(1) $$STD=\sqrt{\frac{1}{N-1}\sum_{n=0}^{N-1}\overset{\text{-}}{x}[n{]}^{2}}$$

where $\overset{\text{-}}{x}[n]$ is the signal after DC removal, and N is the number of sampling points.

Considering that interfacial reflections and energy coupling variations may influence extreme amplitudes, the maximum (Max) and minimum (Min) values of the original signal are also adopted as amplitude-scale descriptors.

Furthermore, given the multimodal nature of dense-state responses with varying peak superposition, extreme values and dispersion alone are insufficient to describe waveform complexity. A waveform descriptor C, defined as the ratio of peak density to zero-crossing density, is therefore introduced:

(2) $$C=\frac{N_{1}}{\max(N_{2},1)}$$

where $N_{1}$ is the number of peaks detected in $\overset{\text{-}}{x}[n]$, and $N_{2}$ is the total number of positive zero-crossings after mean removal.

This parameter characterizes peak sharpness and local superposition without dependence on specific peak locations; larger values of C indicate denser peaks and more complex modal interactions.

(2) Impact response strength

In non-stationary impact signals, single-point extrema are sensitive to noise, and spectral peaks may be unstable under multimodal conditions. To provide a more robust representation of both amplitude and duration, an impact response strength indicator $A_{i}(t)$ is introduced as a discriminative parameter. The expression for impact response strength is given by Equation (3).

(3) $$\begin{array}{ll} A_{i}(t) & =\int |x[n]|dt\\ & =\int_{0}^{t}\left|\sin(\omega_{1}t+\varphi_{1})+...+A_{n}\cos(\omega_{n}t+\varphi_{n})\right|dt\\ & \approx \sum_{i=1}^{i=z}|F_{i}|\times \Delta t \end{array}$$

where F represents the waveform amplitude, Δt is the sampling time interval, and i denotes the sampling point index within time t. This metric complements amplitude extrema and spectral peaks and is particularly suitable for capturing short-term energy amplification and extended attenuation induced by micro-voids.

(3) Amplitude normalization and normalized statistical metrics

In practical applications, variations in impact energy and sensor coupling introduce amplitude-scaling effects. To mitigate such variability while preserving waveform morphology, maximum absolute value normalization is applied. This approach reduces scaling effects and enhances sensitivity to waveform shape and energy distribution. Based on normalized signals, four statistical metrics are computed: Norm_Max (maximum value), Norm_Min (minimum value), Norm_STD (standard deviation), and Impulse_Strength_Norm (normalized impulse response strength).

(4) Spectral peak structure features

As discussed in Section 2.2.1, interfacial variations are primarily reflected in dominant frequency ranges, secondary peak structures, and peak prominence. To avoid reliance on a single dominant peak, the frequencies and amplitudes of the three most significant spectral peaks are extracted. Peaks are identified from the amplitude spectrum and ranked in descending order, forming the feature set $\{Peak_{p}\_Freq,Peak_{p}\_Amp{\}}_{p=1}^{3}$. This representation reduces dimensionality while preserving critical spectral information and enhances discrimination in regions with overlapping spectral patterns.

(5) Sub-band energy and total energy

Micro-void effects are typically expressed as energy redistribution across frequency bands rather than simple peak shifts. To capture this behavior, sub-band energies are computed for the intervals 0–500 Hz, 500–1500 Hz, 1500–2500 Hz, and 2500–4500 Hz. This partitioning aligns with dominant frequency ranges while retaining high-frequency components to improve sensitivity to scattering effects. The energy of the b-th frequency band is defined as

(4) $$E_{b}=\sum_{k:f_{k}\in [f_{b},f_{b+1})}A[k{]}^{2},b=1,\ldots ,4,$$

where A[k] denotes the single-sided amplitude spectrum, $\left[f_{b},f_{b+1}\right)$ represents the b-th frequency band, $f_{k}$ is the frequency point, and b is the band energy index.

The total energy is subsequently defined as:

(5) $$Total\_Energy=\sum_{b=1}^{4}E_{b}$$

A sensitivity analysis using coarser frequency-band schemes obtained by merging adjacent bands showed that the original four-band partition retained the highest Void recall and was therefore adopted for the recall-prioritized screening objective.

(6) Normalized sub-band energy and normalized total energy

Given that absolute energy remains sensitive to excitation intensity, the same sub-band energies are computed on normalized signals, yielding Energy_Norm_0–500 Hz, Energy_Norm_500–1500 Hz, Energy_Norm_1500–2500 Hz, Energy_Norm_2500–4500 Hz, and Total_Energy_Norm. These features emphasize relative energy distribution and improve robustness against operational variability.

**Figure 5:**
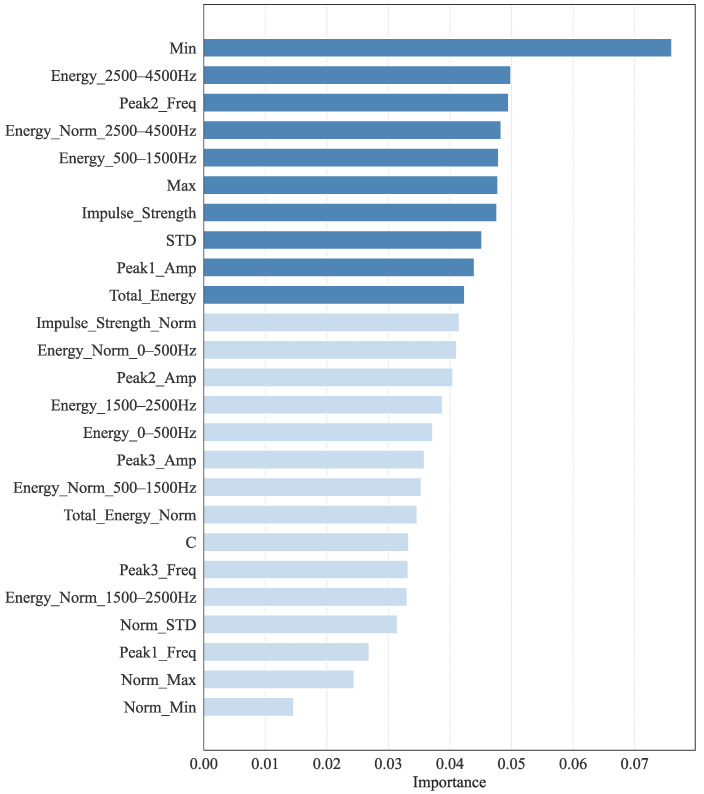
Ranking of the 25 features based on importance scores evaluated by Random Forest.

Although the above features collectively characterize interfacial variations from multiple perspectives, using all features may increase computational cost and reduce model generalization due to redundancy and correlation. Therefore, feature dimensionality reduction is performed to identify the most sensitive parameters. A Random Forest-based feature importance analysis [47], is applied, using 25 extracted features as inputs and three interfacial state labels (dense, slight void, and severe void) as outputs. The ranking results are shown in Figure 5, indicating significant differences in feature contributions. Based on this ranking, the top 10 features (highlighted in dark blue) are selected as the final sensitive feature set for model input. These features integrate time-domain and frequency-domain information, effectively capturing variations in amplitude, fluctuation, dispersion, sub-band energy distribution, and spectral structure. Detailed descriptions and physical interpretations are provided in Table 2.

## 3. Attention Mechanism-Based MLP Model

### 3.1. Overall Framework

**Figure 6:**
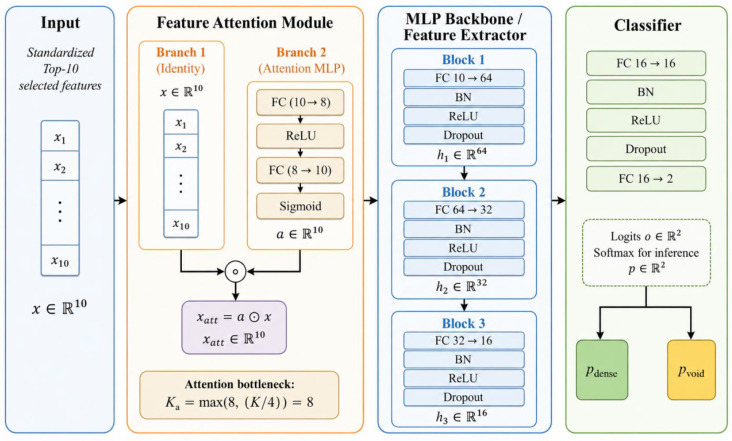
Architecture of the attention mechanism-based MLP model.

The architecture of the lightweight identification model proposed in this study, which integrates a feature attention mechanism with a Multilayer Perceptron (MLP), is illustrated in Figure 6. The model comprises five primary components: an input layer, a feature attention gating module, an MLP backbone for feature extraction, a classification head, and an output decision layer. The operational procedure is as follows: first, structured features obtained during the signal analysis stage are used as model inputs; second, the attention gating module learns feature importance weights specific to each sample and performs dimension-wise recalibration; third, the attention-weighted features are fed into the MLP backbone network, where multi-layer nonlinear transformations are employed to capture high-order interactions among features; finally, the model outputs prediction probabilities for two classes—Dense and Void—and applies a threshold to determine the compactness state.

Compared with end-to-end approaches operating directly on raw time-series signals, the proposed method offers two key advantages. First, the input stage retains the interpretability of mechanism-informed features, allowing the model’s discriminative basis to be associated with physical processes such as frequency-band energy redistribution, spectral peak variations, and impact response intensity. Second, the integration of the attention gating mechanism with the MLP backbone provides enhanced nonlinear modeling capability for complex samples with subtle inter-class differences. This enables the model to learn discriminative boundaries sensitive to micro-voids even under limited sample conditions. As the primary objective is to minimize missed detections of micro-voids rather than merely optimize overall accuracy, the proposed framework constitutes a risk-sensitive identification model oriented toward early warning applications.

### 3.2. Input Standardization and Dimensionality Reduction Strategy

This study utilizes a dataset comprising 705 valid samples. To support early warning of micro-voids at the steel–concrete interface, a void height threshold of 2 mm is defined to establish a binary classification scheme: measurement points corresponding to grid cells with void height (h) greater than 2 mm are labeled as the Void class, whereas those with h ≤ 2 mm are labeled as the Dense class.

To ensure stability during model training, data preprocessing and consistency checks were conducted following feature matrix construction. Missing values in certain features were imputed using column-wise means. Furthermore, residual non-numeric entries or anomalous numerical values were corrected to prevent unstable gradients or abnormal loss fluctuations during training. Standardization parameters were computed exclusively from the training set and subsequently applied to the validation set to avoid information leakage. Given that the selected Top-10 features differ significantly in scale and magnitude, direct training could bias the model toward features with larger numerical ranges, thereby degrading performance. Accordingly, input features were standardized. Let the original feature vector be $x\in R^{K}$; the standardized form is defined as

(6) $$\tilde{x}=\frac{x-\mu }{\sigma }$$

where μ and σ denote the mean and standard deviation of each feature dimension in the training set, respectively. Since these parameters are derived solely from the training data, the validation sets are evaluated within a consistent feature space, ensuring comparability of model outputs.

### 3.3. Feature Attention Gating Network

To enhance sensitivity to critical feature information—particularly addressing the high variability within dense-state signals and the class overlap induced by weak perturbations from micro-voids—a feature attention gating module is introduced immediately after the input layer. This module learns a sample-dependent weight vector to perform adaptive recalibration across feature dimensions, emphasizing sensitive features while suppressing unstable ones. Let $\tilde{x}\in R^{K}$ denote the standardized feature vector after Top-K selection. The attention sub-network takes $\tilde{x}$ as input and produces a weight vector of the same dimension, $a(\tilde{x})\in$(0, 1)^K^. This transformation is formulated as a two-layer feed-forward network augmented with nonlinear mapping:

(7) $$a(\tilde{x})=\sigma \left(W_{2}\phi (W_{1}\tilde{x}+b_{1})+b_{2}\right)$$

where ϕ(·) denotes the ReLU activation function, and $\sigma \left(\cdot \right)$ represents the Sigmoid function that constrains the weights to the interval (0, 1). $W_{1}$ and $W_{2}$ are weight matrices, and $b_{1}$ and $b_{2}$ are bias terms. Subsequently, gating is implemented via element-wise multiplication:

(8) $$x\prime =\tilde{x}⊙a(\tilde{x})$$

where ⊙ signifies the Hadamard product.

The key advantage of this mechanism lies in its ability to adaptively adjust feature importance according to the specific characteristics of each sample. Features exhibiting low stability or high sensitivity to operational variability are down-weighted, whereas features strongly correlated with interfacial defects and exhibiting higher robustness are emphasized. Consequently, weak discriminative information associated with millimeter-scale micro-voids—reflected in localized frequency-band energy, spectral peak structures, and extreme-value statistics—is enhanced, while interference from dense-state variations and noise is suppressed. This improves classification robustness under strong feature overlap and enhances void detection capability.

### 3.4. MLP Backbone Network and Threshold-Based Early Warning Decision

Following the attention gating module, an MLP is employed as the discriminative backbone network. The backbone adopts a three-stage progressive dimensionality reduction architecture. Each stage consists of a Fully Connected (FC) layer, Batch Normalization (BN), a Rectified Linear Unit (ReLU) activation function, and a Dropout layer, designed to improve training stability and mitigate overfitting under limited sample conditions. Denoting the input to the *l*-th layer as $h_{l}$, the output is given by

(9) $$h_{l+1}=Dropout\left(\phi \left(BN\left(W_{l}h_{l}+b_{l}\right)\right)\right),$$

where $W_{l}$ and $b_{l}$ represent the weight matrix and bias vector of the *l*-th layer, respectively, BN(⋅) stabilizes training and accelerates convergence, and Dropout(⋅) reduces overfitting and improves generalization.

Following the backbone, a classification head further transforms the learned representations. This component also incorporates BN, ReLU, and Dropout layers to generate logits for two classes. Following the application of the Softmax function, the probability outputs for the “dense” and “void” classes are obtained as

(10) $$p=Softmax\left(o\right)=\left[p_{0},p_{1}\right],$$

where $p_{1}$ denotes the probability of the “void” class and serves as the basis for the subsequent threshold-based early warning strategy. To balance model capacity and trainability, the hidden-layer dimensions were selected as [64, 32, 16] following a comparison of candidate lightweight architectures using stratified five-fold cross-validation with an inner validation subset. The selection considered Void recall, balanced accuracy, ROC-AUC, PR-AUC, fold-to-fold stability, and model complexity. As shown in Table 3, the [64, 32, 16] configuration achieved the highest mean balanced accuracy, Void recall, ROC-AUC, and PR-AUC among the tested architectures, while also showing the lowest standard deviation in balanced accuracy across folds. Given the recall-prioritized objective of reducing missed detections of micro-voids, the [64, 32, 16] configuration, containing 4052 trainable parameters, was retained as the final model. The attention MLP adopts a (K → max(8, (K/4]) → K) configuration. For the present (K = 10) input features, the attention bottleneck dimension was therefore set to 8. This configuration was intended to provide sufficient feature-recalibration capacity while limiting the number of trainable parameters.

Unlike conventional classification tasks that adopt a fixed threshold of 0.5, this study treats the decision threshold as an integral component of the early warning strategy. Given that the objective is to minimize missed detections of micro-voids, the threshold τ is scanned over the interval [0.05, 0.95] with a step size of 0.01 on the validation set. The optimal threshold is selected based on a joint consideration of classification accuracy and early warning performance. Results indicate that the model achieves optimal validation accuracy at τ=0.33. Accordingly, the final decision rule is defined as

(11) $$\hat{y}=\left\{\begin{array}{ll} 1, & p_{1}\geq 0.33\\ 0, & p_{1}<0.33 \end{array}\right.$$

The fact that this threshold is significantly lower than 0.5 indicates that a decision boundary biased toward void detection is more appropriate for micro-void identification. From an engineering perspective, this implies that samples with ambiguous characteristics are preferentially classified as potential voids to reduce the risk of missed detections. Given that the signal differences associated with millimeter-scale voids, such as variations in sub-band energy and local peak structures, are often subtle, a conventional 0.5 threshold may lead to high-risk samples being incorrectly classified as dense due to marginally lower probabilities, thereby undermining the effectiveness of the early warning system.

## 4. Results Analysis and Discussion

### 4.1. Training and Testing

#### 4.1.1. Dataset Partitioning

Given that full-scale model experiments do not presuppose the presence of void defects, the casting procedures and self-compacting concrete materials employed are consistent with those used in actual engineering practice. Consequently, void defects occur as low-probability events, leading to a dataset in which samples from dense regions significantly outnumber those from defective regions. To mitigate the influence of random partitioning and ensure consistency in class proportions between training and validation sets, a stratified sampling strategy was adopted with an 85%:15% split ratio and a fixed random seed of 42. The resulting training set contains 599 samples (336 dense and 263 void), while the validation set comprises 106 samples (60 dense and 46 void). This distribution is consistent with the overall dataset characteristics, thereby preserving statistical reliability for evaluating void detection performance under limited sample conditions. Furthermore, the class proportions in the training set are maintained through stratified sampling, ensuring that gradient contributions during training are aligned with validation evaluation, thereby enhancing the reliability of threshold selection and generalization assessment.

#### 4.1.2. Training and Testing Environment

The training and testing environments, network architecture, and primary hyperparameters employed in this study are summarized in Table 4. The final training parameters were selected through repeated trials by jointly considering Void recall, balanced accuracy, weighted F1-score, model complexity, and training stability.

### 4.2. Model Training Results

**Figure 7:**
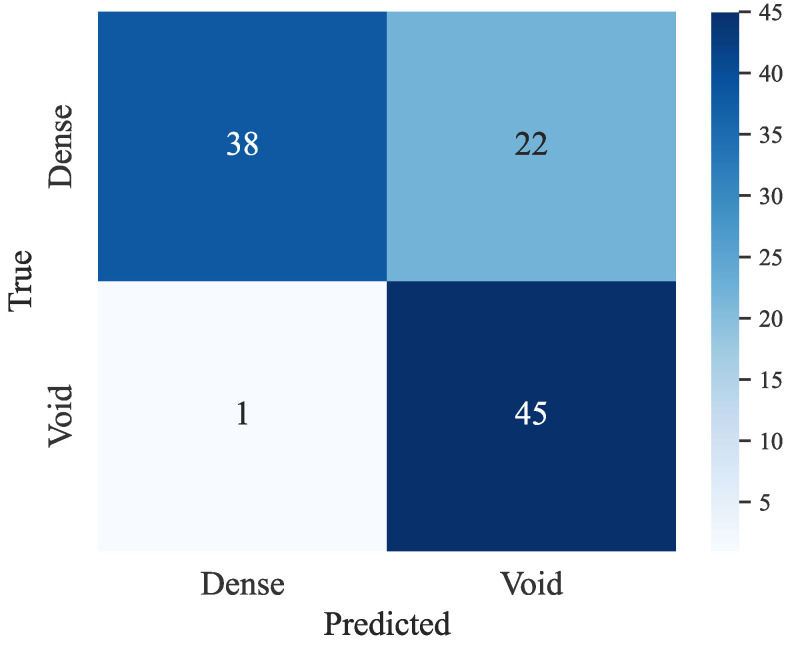
Confusion matrix.

**Figure 8:**
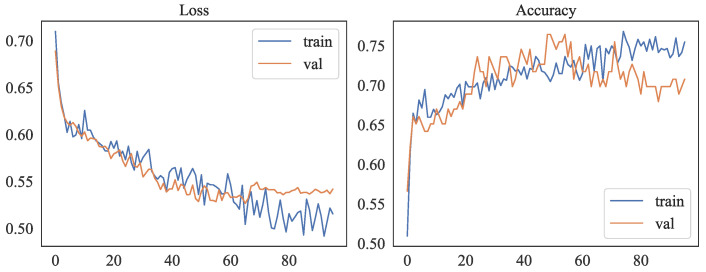
Training curves.

The distribution of validation outcomes across different categories is shown in Figure 7, while the training curves are presented in Figure 8. The results indicate that the proposed Feature Attention MLP exhibits stable convergence behavior. As training progresses, the loss function decreases steadily, and validation accuracy gradually improves to a high level, indicating that the model effectively captures discriminative features associated with micro-voids. In the later stages of training, although performance on the training set continues to improve, validation metrics exhibit certain fluctuations, suggesting a mild tendency toward overfitting. To improve training stability and mitigate overfitting, an adaptive learning rate decay strategy and early stopping mechanism were implemented, with model weights corresponding to the minimum validation loss selected as the final model.

At the recall-prioritized operating point used for the original train–validation split, the final model achieved an accuracy of 78.30% and a weighted F1-score of 0.7802 on the validation subset. The confusion matrix shows that 45 of 46 Void samples were correctly identified, corresponding to a Void recall of 97.8%. However, 22 of 60 Dense samples were classified as Void, indicating that the recall-oriented operating point was accompanied by a non-negligible false-positive screening burden. Therefore, the proposed model is intended to identify candidate locations for subsequent inspection rather than to serve as a standalone diagnostic or maintenance-decision tool.

**Figure 9:**
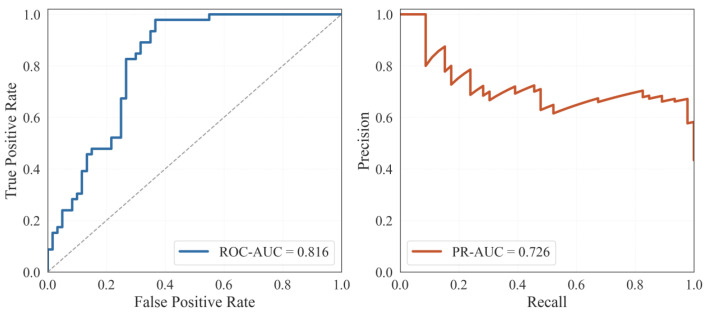
ROC and precision-recall curves.

To further assess threshold-independent discrimination performance, ROC and precision–recall (PR) curves were generated for the validation subset of the original train–validation split, as shown in Figure 9. The gray dotted diagonal line in the ROC curve represents the no-discrimination baseline, corresponding to random classification performance. The ROC-AUC was 0.816, indicating that the predicted probabilities provided meaningful overall separation between Dense and Void samples. The ROC curve also rises above the diagonal reference line, indicating discrimination performance above random classification.

The PR-AUC was 0.726. The PR curve indicates that the model maintained moderate precision across a broad range of recall levels, although precision gradually declined as recall approached 1.0. This trend reflects the inherent trade-off in the present micro-void screening task: relaxing the decision threshold increases the probability of identifying potential Void locations, but also increases the number of Dense samples classified as Void. The ROC and PR results therefore complement the confusion-matrix results by demonstrating both the probability-ranking capability of the model and the associated recall–false-positive trade-off.

### 4.3. Comparative and Ablation Studies

#### 4.3.1. Evaluation Metrics and Statistical Methodology

Considering that this study focuses on early warning of micro-voids, the engineering risk associated with missed detections typically exceeds that of false positives. Therefore, the recall rate for the void class is selected as the primary evaluation metric to quantify the model’s ability to identify potential void risks. To provide a balanced assessment of overall classification performance, the Weighted-F1 score is also reported, reflecting discriminative effectiveness under class imbalance. In addition, Accuracy and Precision are computed to evaluate false positive control.

All experiments follow an identical statistical protocol. Specifically, data are partitioned into training and validation sets using stratified random sampling, with a fixed validation ratio of 15% and a consistent random seed of 42. This ensures comparability across different models.

#### 4.3.2. Baseline Methods

To evaluate the effectiveness of the proposed method for micro-void early warning, it is compared with representative traditional machine learning classifiers. This comparison aims to assess the advantages of deep feature modeling under complex data distributions. Three baseline models are considered: Random Forest (RF), Support Vector Machine (SVM), and Logistic Regression (LR). All baseline models and the proposed method use the same set of 10 input features. To reduce uncertainty from hyperparameter tuning, a practical strategy consistent with engineering applications was adopted, in which only a limited number of key parameters were adjusted while others were retained at default values. Parameter selection was performed on the training set, and final performance was evaluated on the validation set. For SVM and LR, input features were standardized. Although RF is generally insensitive to feature scaling, all features were standardized to ensure consistency across models.

#### 4.3.3. Ablation Study Design

To quantitatively evaluate the contribution of each key component in the proposed model, three ablation experiments were designed and compared with the complete model.

(1) w/o attention: The feature attention gating module was removed, and feature vectors were directly input into an MLP backbone of equivalent scale. This configuration evaluates whether adaptive feature re-weighting mitigates class overlap and misclassification caused by intra-class variability in dense signals.
(2) w/o top-K: The Random Forest-based feature selection step was removed, and all features were used as input to the Attention-MLP model. This experiment examines whether feature dimensionality reduction improves generalization stability, reduces interference from redundant features, and prevents the model from learning noise as discriminative cues.
(3) Varying K: Keeping all other settings unchanged, the effect of different input dimensions was evaluated for K ∈ {5, 10, 15, 20, 25}. A small K may discard weak but relevant diagnostic information, while a large K may introduce redundant or confounding features, increasing false positives.

All ablation experiments were conducted under identical training conditions, including optimizer settings, learning rate scheduling, and early stopping, and were evaluated using consistent metrics and statistical procedures.

#### 4.3.4. Results Analysis

**Figure 10:**
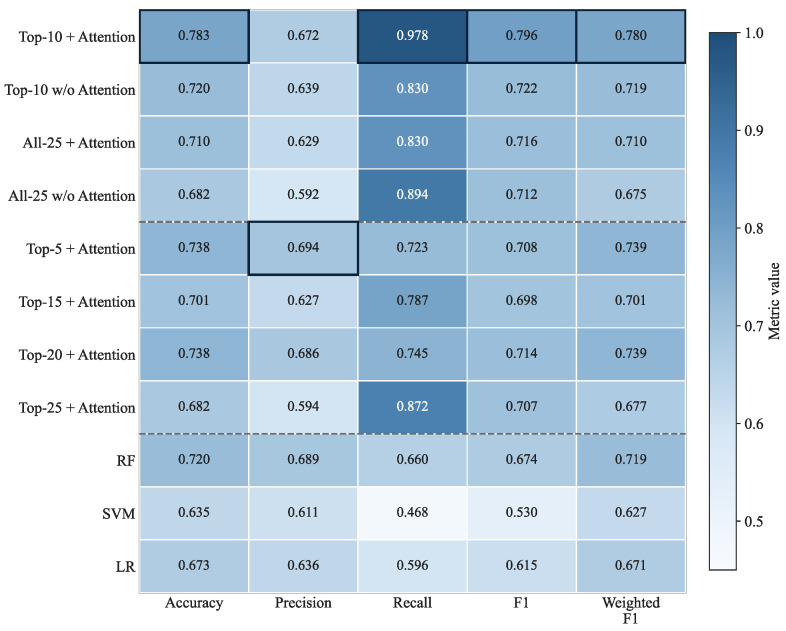
Comprehensive ablation heatmap.

As shown in Table 5 and Figure 10, the final proposed model, denoted as mlp_topk_attention, achieves the highest void recall rate and the best overall performance among all experimental configurations. Specifically, it attains an Accuracy of 0.7830, Void Precision of 0.6716, Void Recall of 0.9783, Void F1-score of 0.7965, and Weighted-F1 of 0.7802.

Compared with traditional baselines, the primary advantage of the proposed model lies in its superior coverage of void samples. While RF achieves higher Precision, indicating better control of false positives, it exhibits lower Void Recall and Weighted-F1, suggesting a more conservative decision boundary that leads to increased missed detections. Similarly, SVM and LR show lower Recall and Weighted-F1 compared with the proposed model, reflecting limited capability in capturing complex nonlinear relationships and handling feature overlap in subtle anomaly detection tasks such as micro-void identification.

The ablation results indicate that the effects of feature attention and threshold selection should be interpreted separately. The comparison in Table 5 shows that the Top-10 model with feature attention achieved better overall performance than the conventional machine-learning baselines and the alternative feature configurations. However, because the threshold settings differ among the compared configurations, the Recall difference between the attention and non-attention models should not be attributed solely to the attention mechanism.

Sensitivity analysis with varying K values highlights the importance of feature dimensionality. At K = 5, Precision is slightly improved, but Recall decreases, indicating that excessive feature reduction leads to loss of critical information and increased missed detections. Increasing K to 15, 20, or 25 does not improve performance; instead, it results in degradation across multiple metrics due to the introduction of redundant or noisy features.

The dedicated attention-threshold ablation analysis further showed that threshold selection substantially governs the trade-off between Void recall and Dense-state specificity, whereas feature attention contributes to feature recalibration and probability discrimination. Therefore, the recall-oriented behavior of the final model arises from the combined effects of Top-K feature selection, feature-attention-based recalibration, nonlinear classification, and threshold selection, rather than from the attention mechanism alone.

#### 4.3.5. Feature-Level Interpretability Analysis

Because the proposed model receives a standardized 10-dimensional feature vector rather than raw waveforms or spectral images, gradient attribution was performed in the feature-input space with the model in evaluation mode. For the i-th validation sample, the sensitivity of the pre-Softmax void-class logit, $o_{void}^{\left(i\right)}$, to feature $z_{ij}$ was quantified as

(12) $$S_{j}=\frac{1}{N}\sum_{i=1}^{N}\left|\frac{\partial o_{void}^{\left(i\right)}}{\partial z_{ij}}\right|$$

where N is the number of validation samples. Mean attention weights were also calculated over the same samples.

As shown in Table 6, which presents the five features with the highest mean gradient saliency, Energy_2500–4500 Hz exhibited the highest gradient saliency, followed by STD, Impulse_Strength, Total_Energy, and Energy_500–1500 Hz. These results indicate that the void prediction was mainly sensitive to high-frequency energy redistribution, waveform fluctuation, transient impact-response strength, and total signal energy, which are physically associated with local wave attenuation, reflection, and energy redistribution at steel–concrete interface micro-voids.

#### 4.3.6. Generalization Performance Evaluation

To reduce the dependence on a single train–validation split, stratified five-fold cross-validation with an inner validation subset was conducted. In each outer fold, the model-development data were divided into inner training and validation subsets for model fitting, early stopping, and threshold selection, while the outer held-out fold was used only for final evaluation. Results are reported as the mean ± standard deviation across the five outer folds.

As summarized in Table 7, the inner-validation-optimized threshold achieved the highest balanced accuracy (0.682 ± 0.021), with a Void recall of 0.845 ± 0.035 and a Dense-state specificity of 0.520 ± 0.054. The recall-first threshold of 0.33 further increased Void recall to 0.906 ± 0.083, but reduced accuracy and Dense-state specificity. Across the five folds, the ROC-AUC and PR-AUC were 0.740 ± 0.049 and 0.661 ± 0.063, respectively.

These results provide a more reliable estimate of internal generalization stability across different stratified data partitions than the original single train–validation split. Overall, the five-fold cross-validation results provide a more reliable estimate of internal performance stability than the original single train–validation split. The fold-optimized threshold offers a relatively balanced operating condition, whereas the threshold of 0.33 is more appropriate for recall-prioritized preliminary screening. Since all samples were obtained from the same full-scale steel shell–concrete structure and experimental campaign, these results should be interpreted as internal validation rather than independent external validation. Furthermore, it should be noted that in actual engineering inspections, interfacial voids are low-probability events. Consequently, applying this recall-prioritized screening model in highly imbalanced field scenarios may result in a lower overall precision (i.e., a higher proportion of false-positive alarms among the predicted voids) than observed in the present experimental dataset.

### 4.4. Engineering Validation

**Figure 11:**
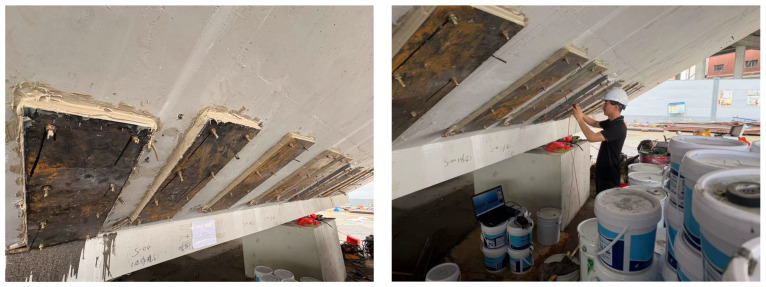
Configuration of the full-scale validation model.

To examine the applicability of the proposed method under changed structural and interface conditions, a full-scale validation model from another engineering project was investigated. As shown in Figure 11, the model consisted of prestressed steel inclined I-beams connected to an existing concrete box girder through an epoxy–resin–mortar interface. Compared with the original steel shell–concrete model, it involved different structural geometries, interface materials, boundary conditions, and load-transfer characteristics.

The processing procedure developed for the original dataset was retained without modification. Impact elastic-wave responses collected from the validation model were converted into the same structured feature representation, and the training-derived normalization parameters, selected Top-10 features, trained Feature-Attention MLP, and decision threshold of τ = 0.33 were directly applied. No retraining, feature reselection, normalization adjustment, or threshold adjustment was performed.

**Figure 12:**
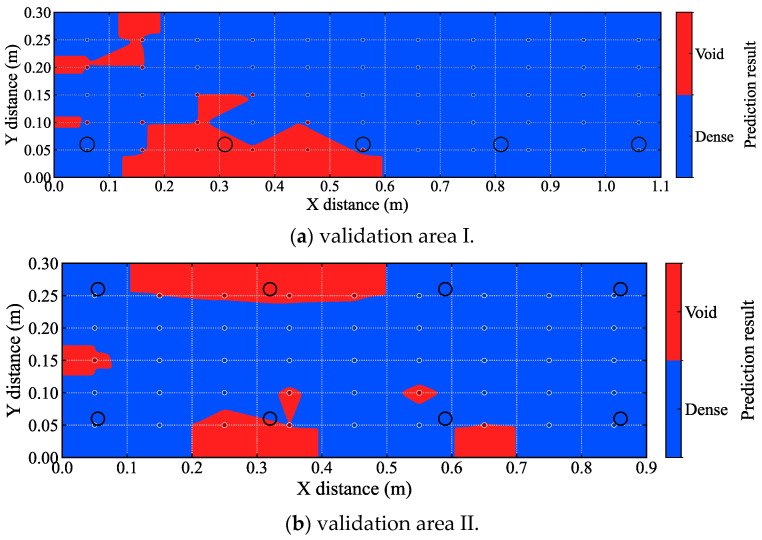
Spatial distribution of predicted Dense and potential Void regions in two full-scale validation areas.

Figure 12 presents the spatial prediction maps for two validation areas. In both areas, the predicted potential Void regions exhibit localized and clustered distributions rather than random scattering, providing an intuitive representation of possible interface-defect regions. Comparison with void-height measurements indicated that more than 90% of the verified Void locations were identified as potential Void regions.

These results provide preliminary evidence that the proposed feature-based screening framework can be transferred to related steel–concrete composite interfaces under changed structural and interface conditions. Nevertheless, further validation using additional full-scale models and more comprehensive verified Dense and Void locations is needed.

## 5. Conclusions

This study addresses the early identification of micro-voids at the thick steel shell–concrete interface, a problem characterized by subtle inter-class differences, significant feature overlap, and high sensitivity to operational conditions. The study systematically analyzed the evolution of impact response signals in both time and frequency domains, constructed a structured feature system for characterizing interface states, and developed an MLP-based identification model integrated with a feature attention mechanism. The identification performance and engineering applicability of the proposed method were validated using data from full-scale model experiments and field inspections conducted at the Shenzhen–Zhongshan Link. The main conclusions and discussions are summarized as follows:

(1) Impact response signals associated with micro-voids at the thick steel shell–concrete interface exhibit pronounced intra-class variability and inter-class overlap. Raw signals corresponding to different dense states show substantial diversity, with a single state capable of presenting multiple representative time–frequency response patterns. Simultaneously, considerable overlap exists among different states, particularly in terms of dominant peak locations, peak number configurations, and energy distribution characteristics. These observations indicate that reliance on a single peak location or a limited set of empirical spectral criteria is insufficient for achieving reliable discrimination during the early stages of micro-void formation.
(2) The selected Top-10 features effectively capture local response variations induced by interfacial micro-voids. Based on raw impact-echo signals, a multidimensional feature system was established, incorporating time-domain statistical features, spectral peak structure features, sub-band energy features, and normalized energy features. Random Forest importance analysis indicates that Min, Energy_2500–4500 Hz, Peak2_Freq, Energy_Norm_2500–4500 Hz, Energy_500–1500 Hz, Max, Impulse_Strength, STD, Peak1_Amp and Total_Energy significantly contribute to interface state identification. This demonstrates that discriminative information related to micro-voids is primarily distributed across multiple dimensions, including energy redistribution within specific frequency bands, variations in extreme-value statistics, and modifications in spectral peak structures.
(3) Under the original train–validation split, the proposed Feature-Attention MLP achieved a Void recall of 0.978, with 45 of 46 Void samples correctly identified. This result indicates that the framework provides strong recall-prioritized screening capability for micro-voids under the investigated full-scale steel shell–concrete conditions.

Although the proposed method has been preliminarily validated using full-scale experimental and independent validation data, further work is required to improve its robustness and broader applicability. Future studies should include additional steel–concrete structures under varied material, interface, construction, and service conditions, together with repeated impacts, sensor-coupling control, and signal-quality assessment. Furthermore, while the model demonstrated robustness against moderate feature-level perturbations, its performance under physical acoustic interference directly injected into raw impact waveforms remains to be systematically investigated in future studies. The adopted 2 mm criterion is a void-height decision threshold rather than a lateral spatial-resolution limit; thus, the method is intended for point-wise screening rather than sub-millimeter boundary reconstruction, and near-threshold or boundary locations require denser measurements and local verification. Multi-sensor fusion with complementary modalities, such as ultrasonic testing and thermal imaging, should also be investigated. Ultimately, the framework should support a staged workflow of recall-prioritized screening, repeated measurement and localized confirmation, followed by engineering intervention only after complementary NDT or cover-opening verification where feasible.

## Figures and Tables

**Table 1:** Classification of void defect severity.

Defect Type	Dense	Slight Void	Severe Void
Void depth (d)	d ≤ 2 mm	2 mm < d ≤ 3 mm	d > 3 mm

**Table 2:** Summary of extracted 10-dimensional features.

	Feature Category	Feature Name	Physical Meaning
1	Time-domain	Max	Represents the positive peak amplitude of the signal, reflecting the maximum positive excitation level of the impact response.
2	Time-domain	Min	Represents the negative peak amplitude of the signal, indicating the intensity of the reverse response and local fluctuation characteristics of the waveform.
3	Time-domain	STD	Represents the overall dispersion and fluctuation intensity of the signal, reflecting the stability of the response and the level of energy variation.
4	Time-domain	Impulse Strength	Represents the comprehensive intensity of the transient impact response, indicating the enhanced local vibration characteristics under defective conditions.
5	Frequency-domain	Peak1_Amp	Represents the amplitude of the primary peak, reflecting the energy intensity of the dominant frequency component.
6	Frequency-domain	Peak2_Freq	Represents the frequency position of the secondary peak, indicating changes in the secondary spectral structure and modal differentiation characteristics.
7	Frequency-domain	Total_Energy	Represents the total energy across the entire frequency domain, reflecting the overall response energy level.
8	Frequency-domain	Energy_500–1500 Hz	Represents the energy distribution within the primary characteristic frequency band, indicating the influence of interface state changes on the mid-frequency response.
9	Frequency-domain	Energy_2500–4500 Hz	Represents the energy level in the high-frequency band, reflecting local stiffness anomalies or variations in high-frequency-sensitive components.
10	Frequency-domain	Energy_Norm_2500–4500 Hz	Represents the normalized energy proportion in the high-frequency band, indicating the relative contribution of high-frequency components to the overall spectrum.

**Table 3:** Nested stratified five-fold cross-validation comparison of candidate network architectures.

Architecture	Trainable Parameters	Accuracy	Balanced Acc.	Void Recall	Dense-State Specificity	ROC-AUC	PR-AUC
[32]	1188	0.677 ± 0.045	0.687 ± 0.041	0.774 ± 0.079	0.601 ± 0.104	0.728 ± 0.056	0.649 ± 0.070
[64, 32]	3748	0.658 ± 0.046	0.668 ± 0.047	0.748 ± 0.087	0.588 ± 0.070	0.726 ± 0.055	0.639 ± 0.078
[64, 32, 16]	4052	0.670 ± 0.018	0.689 ± 0.017	0.851 ± 0.040	0.528 ± 0.047	0.746 ± 0.042	0.667 ± 0.059
[128, 64, 32]	12,964	0.648 ± 0.039	0.664 ± 0.031	0.793 ± 0.092	0.536 ± 0.121	0.732 ± 0.051	0.666 ± 0.070

**Table 4:** Model training environment and primary parameter settings.

Parameter	Setting
Operating system	Windows 11
Computation mode	CPU
Attention module	Fully connected layer + sigmoid activation
Classifier structure	Three hidden layers
Hidden layer dimensions	64, 32, 16
Hidden layer components	Batch Normalization + ReLU + Dropout
Optimizer	AdamW
Initial learning rate	0.001
Weight decay coefficient	1 × 10^−4^
Training batch size	32
Validation batch size	64
Maximum training epochs	200
Learning rate scheduling strategy	ReduceLROnPlateau
Decay factor	0.5
Patience rounds for learning rate scheduling	6
Minimum learning rate	1 × 10^−5^
Patience rounds for early stopping	30

**Table 5:** Experimental results across different models and feature configurations.

Experiment	Use_Attention	Top_k	Input_dim	Threshold	Accuracy	Precision_pos	Recall_pos	f1_pos	f1_Weighted
mlp_topk_attention	TRUE	10	10	0.33	0.7830	0.6716	0.9783	0.7965	0.7802
mlp_topk_no_attention	FALSE	10	10	0.36	0.7196	0.6393	0.8298	0.7222	0.7193
mlp_all_attention	TRUE	0	25	0.39	0.7103	0.6290	0.8298	0.7156	0.7095
mlp_all_no_attention	FALSE	0	25	0.3	0.6822	0.5915	0.8936	0.7119	0.6748
mlp_topk_5	TRUE	5	5	0.46	0.7383	0.6939	0.7234	0.7083	0.7388
mlp_topk_15	TRUE	15	15	0.37	0.7009	0.6271	0.7872	0.6981	0.7012
mlp_topk_20	TRUE	20	20	0.45	0.7383	0.6863	0.7449	0.7143	0.7391
mlp_topk_25	TRUE	25	25	0.25	0.6822	0.5942	0.8723	0.7069	0.6767
RF		10	10		0.7196	0.6889	0.6596	0.6739	0.7189
SVM		10	10		0.6355	0.6111	0.4681	0.5301	0.6267
LR		10	10		0.6729	0.6364	0.5957	0.6154	0.6715

**Table 6:** Mean gradient saliency, mean attention weight, and physical interpretation of the selected features on the held-out validation subset.

Feature	Mean Absolute Gradient Saliency	Mean Attention Weight	Physical Interpretation
Energy_2500–4500 Hz	2.285	0.592	High-frequency energy redistribution and attenuation
STD	1.304	0.589	Waveform fluctuation intensity
Impulse_Strength	1.264	0.561	Transient impact response strength
Total_Energy	1.158	0.480	Overall response energy
Energy_500–1500 Hz	0.911	0.568	Mid-frequency energy redistribution

**Table 7:** Internal generalization performance under different threshold policies based on stratified five-fold cross-validation.

Threshold Policy	Threshold	Accuracy	Balanced Accuracy	Void Precision	Void Recall	Dense Specificity
Fixed threshold	0.50	0.661 ± 0.039	0.668 ± 0.040	0.598 ± 0.043	0.725 ± 0.143	0.611 ± 0.124
Inner-validation-optimized threshold	0.422 ± 0.077	0.662 ± 0.023	0.682 ± 0.021	0.580 ± 0.020	0.845 ± 0.035	0.520 ± 0.054
Recall-first threshold	0.33	0.594 ± 0.048	0.629 ± 0.035	0.526 ± 0.042	0.906 ± 0.083	0.351 ± 0.145

## Data Availability

The original contributions presented in this study are included in the article. Further inquiries can be directed to the corresponding author.
